# Epidemiology of viral disease outbreaks in Odisha, India (2010–2019)

**DOI:** 10.1017/S0950268820001594

**Published:** 2020-07-16

**Authors:** J.S. Kshatri, J. Turuk, J. Sabat, S. Subhadra, L.M. Ho, S. Rath, S.K. Palo, D. Bhattacharya, B. Dwibedi, S. Pati

**Affiliations:** 1 Indian Council of Medical Research-Regional Medical Research Centre, Bhubaneswar, Nalco Square, Odisha-751023, India; 2 All India Institute of Medical Sciences, Bhubaneswar, India

**Keywords:** Outbreaks, Surveillance, Virology (human) and epidemiology

## Abstract

Despite consistent public health efforts, the burden of viral disease in India remains high. The present study was undertaken to understand the aetiology, frequency and distribution of viral disease outbreaks in the state of Odisha between 2010 and 2019. This was a prospective study conducted at the Virology Research and Diagnostic Laboratory located at ICMR-Regional Medical Research Centre, Bhubaneswar, Odisha, wherein all the outbreaks of viral aetiologies were investigated and analysed to provide a comprehensive picture of the state of viral disease outbreaks in the region. A total of 191 suspected viral outbreaks were investigated by the team from VRDL during September 2010 and September 2019 reported from all the 30 districts of Odisha. Annual number of suspected cases ranged from 185 to 1002. The most commonly suspected outbreaks were of viral hepatitis (55 outbreaks; 1223 cases) followed by dengue (45 outbreaks; 1185 cases), chickenpox (30 outbreaks; 421 cases), viral encephalitis (27 outbreaks; 930 cases), measles (23 outbreaks; 464 cases), chikungunya (10 outbreaks; 593 cases) and rubella (1 outbreak; 60). The outbreaks peaked in frequency and intensity during the months of July and September. The epidemiology of viral disease outbreaks in the region is presented in the study. Health system preparedness based on evidence is essential for early detection and adequate response to such viral outbreaks.

## Introduction

Infectious diseases remain a major contributor to the global, regional and national burden of mortality and disabilities [1, 2]. Viral diseases are gaining importance due to the emergence of new pathogens, improved surveillance and diagnostic capacity, rapid transmission and limited therapeutic measures available. Viral outbreaks are of particular public health concern as they tend to be unpredictable in occurrence and of unknown origin; and warrant immediate effective control measures to avoid high morbidity and mortality.

A disease outbreak is defined as the occurrence of clusters of epidemiological connected cases, which often requires extra human and financial resources and may also rely on additional partners, agencies and other sectors [3, 4].

Reports from the past two decades have shown a significantly increasing frequency and burden of viral disease outbreaks globally [5, 6]. The weekly Integrated Disease Surveillance Programme (IDSP) Outbreak Surveillance which documents and reports any disease outbreak in India revealed a steep increase in the frequency and variety of outbreaks across India. The absolute number of reported outbreaks increased from 553 in 2007 to 1714 in 2017 [7]. Similarly, an increase has also been reported from eastern India as well as the state of Odisha [8–11].

Response to such viral outbreaks needs a comprehensive and interdisciplinary team approach. Health system mechanisms and preparedness for detection, confirmation and control of such events are of paramount importance. To strengthen these efforts, the Department of Health Research, Ministry of Health, Government of India, has set up a network of Viral Research and Diagnostic Laboratories (VRDL) across India that have been working for the diagnosis of different viruses from suspected human cases. In Odisha, VRDL has been working towards viral diagnosis since 2010 and investigations have been standardised for more than 50 different viruses which include serological tests, molecular investigations (conventional PCR and real-time PCR), sequencing and culture.

Knowledge of the frequency, distribution, aetiologies and determinants of outbreaks would be a useful tool for preparedness and planning appropriate intervention measures to control the same. While there are existing case reports and analyses of individual outbreaks in the country and state, there are no comprehensive research studies on multiple suspected outbreaks investigated using a standardised method. With this background, the present study was undertaken to understand the aetiology, frequency and distribution of viral disease outbreaks in the state of Odisha between 2010 and 2019.

## Methodology

This prospective study was conducted at the VRDL located at Indian Council of Medical Research's Regional Medical Research Centre at Bhubaneswar, Odisha (ICMR-RMRC). The study was carried out in the state of Odisha in eastern India with a population of 46 million people, economic indices below the national average, and a large indigenous or tribal population (22%) [12,13]. The study period was between September 2010 and September 2019. Study participants were all cases of specific suspected viral syndromes reported across the state that were classified to be outbreaks and investigated by the study team.

Outbreak investigation methods: The Regional VRDL conducts sporadic sample testing as well as outbreak investigations in the state of Odisha and parts of neighbouring states. VRDL has a standing Rapid Response Team (RRT) comprising of a roster of epidemiologists, entomologists, microbiologists, clinicians, lab technicians and support staff. Following the information on suspected outbreaks of infectious diseases through an established network with the state health department, IDSP and/or media reports, outbreak response and investigations were carried out by the RRT. Investigations of the outbreaks were done following a standard 10-step protocol developed by ICMR-National Institute of Epidemiology (NIE) for field epidemiology [14]. Suitable samples were collected for laboratory confirmation and transported following international guidelines adopted by the IDSP [15]. Laboratory investigations for the samples were carried out using syndromic diagnostic algorithms for five syndromes developed by the ICMR-National Institute of Virology (NIV), India, as given below:

Data management and reporting: The clinical information and patient identifiers were collected from the suspected cases in the specified Clinical Recruitment Format (CRF) and were entered online through an online portal maintained by NIE. The laboratory investigation reports were shared with the state health department at the local and state level through telephone, e-mail, fax and postal media as soon as possible for public health action. Control measures for the outbreaks were suggested to the state health department according to IDSP guidelines and their implementation monitored.

Data analysis: Data were downloaded from the online portal in excel format and analysed for deriving descriptive statistical measures using SPSS ver. 23. Tables and some of the graphs were generated using modules of R-software package for statistical analysis. Geospatial analyses were done using the QGIS (ver.3.4) software.

Ethical concerns: Approval was obtained from the Institutional Human Ethical Committee of ICMR RMRC, Bhubaneswar, for the study. Data confidentiality was ensured and only aggregated patient data are being presented in the analyses. Informed consent was obtained from the participants, following the ICMR National Ethical Guidelines for biomedical research involving human subjects [16].

## Results

A total of 191 suspected viral outbreaks were investigated by the team from VRDL during September 2010 and September 2019. These outbreaks were reported from all 30 districts of Odisha. A total of 5486 persons were included in these investigations, among them 2770 (50.5%) were females and 2716 (49.5%) were males. The mean age of the study participants was 26.7 years (s.d. = 18.3 years) and majority of the participants were adults between 18 and 60 years of age (2890; 52.7%) followed by children between 5 and 13 years (1204; 21.9%) and children under the age of 5 years (578; 10.5%). The study also included 21 infants, 537 adolescents (13–18 years) and 256 elderly aged over 60 years.

**Fig. 1.**
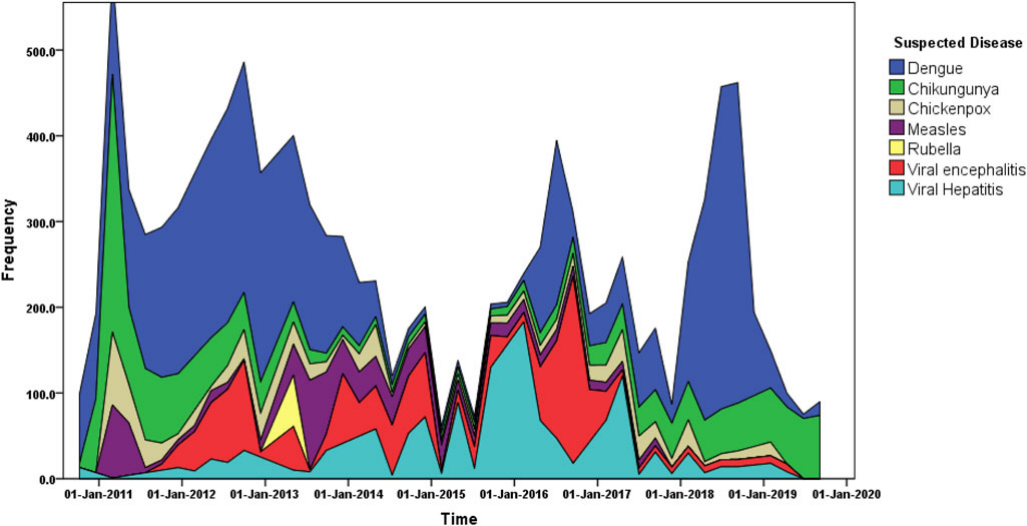
Time series distribution of suspected viral outbreak cases between 2010 and 2019 in the state of Odisha.

The annual number of suspected cases ranged from 185 to 1002. The most commonly suspected outbreaks were of viral hepatitis (55 outbreaks; 1223 cases), followed by dengue (45 outbreaks; 1185 cases), chickenpox (30 outbreaks; 421 cases), viral encephalitis (27 outbreaks; 930 cases), measles (23 outbreaks; 464 cases), chikungunya (10 outbreaks; 593 cases) and rubella (1 outbreak; 60). The temporal time trends of these outbreaks are given in Figure 1.

**Fig. 2.**
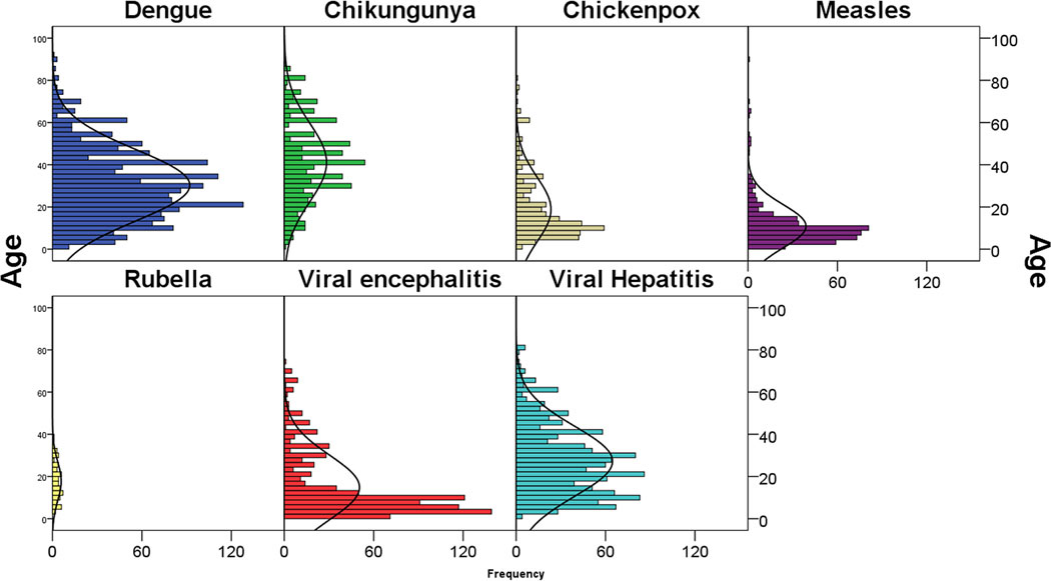
Age distribution of the suspected viral outbreak cases between 2010 and 2019 in the state of Odisha.

The outbreaks were reported throughout the year but maximum cases were reported in the month of July followed by September (Figure 2).

The mean age of the suspected cases for dengue was 30.3 years (95% CI 29.5–31.1 years), chikungunya was 41.1 years (95% CI 39.6–42.6 years), chickenpox was 18.8 years (95% CI 17.3–20.3 years), measles was 10.9 years (95% CI 9.9–11.8 years), rubella was 17.7 years (95% CI 15.4–20.1 years), viral encephalitis was 14.8 years (95% CI 13.8–15.8 years), viral hepatitis was 27.2 years (95% CI 26.3–28.1 years).

The most common sample collected was blood/serum (98.5%), but samples such as throat swabs, cerebrospinal fluid (CSF), urine, stool, vesicular fluid/swabs were also collected depending on the suspected disease outbreak. The results of these tests are summarised below in Tables 1 and 2.

**Table 1.** Syndromic approach for investigations of suspected viral outbreaks in the state of Odisha reported between 2010 and 2019

Syndrome	Case definitions	Recommended samples	Diseases investigated	Techniques used
Acute febrile illness with rash	A fever of 38 °C or higher at presentation or history of fever that persisted for 2–7 days with no localizing source and maculopapular rash	Acute phase serum/plasma	Measles Rubella Chickenpox Chikungunya Dengue	IgM ELISA RT-PCR (for serotype and genotype)
Acute respiratory syndrome	Acute respiratory infection (ARI): sudden onset of respiratory infection symptoms (cough, sore throat shortness of breath, coryza) Severe ARI: ARI with history of fever or measured fever of ⩾38 C°; and cough; with onset within the last 10 days; and requires hospitalisation	Nasopharyngeal swabs, throat swabs, nasal aspirate (with cold chain)	Influenza A and B, H1N1, H3N2	Multiplex RT PCR
Viral hepatitis	An acute illness typically including acute jaundice, dark urine, anorexia, malaise, extreme fatigue and right upper quadrant tenderness. Biological signs include increased urine urobilinogen and >2.5 times the upper limit of serum alanine aminotransferase	Serum/plasma	HAV HEV	IgM ELISA RT-PCR
Viral encephalitis	Suspected case: Acute onset of fever, not more than 5–7 days duration Change in mental status with/without: New onset of seizures (excluding febrile seizures) (Other early clinical findings – may include irritability, somnolence or abnormal behaviour greater than that seen with usual febrile illness)	CSF blood/serum, Plasma	Dengue, JE, Chikungunya,	IgM-ELISA

Legend: HB/C/E/AV, JE, IgM, ELISA, PCR, RTPCR, RDK, DNAPCR, RNAPCR.

**Table 2.** Results of the diagnostic tests for the suspected cases of viral outbreaks between 2010 and 2019 in the state of Odisha

Suspected disease	Result	Varicella		Measles		Rubella	Dengue		CHIKV	JEV		HSV			HAV		HEV	
		IgM	PCR	IgM	PCR	IgM	IgM	NS1	IgM	IgM	PCR	HSV1-IgM	HSV2-IgM	HSV-PCR/RT-PCR	IgM	PCR	IgM	PCR
Chickenpox	Positive	264	2	32	0	–	–	–	–	–	–	–	–	–	–	–	–	–
	Negative	150	39	49	4	–	–	–	–	–	–	–	–	–	–	–	–	–
	Equivocal	7	–	–	–	–	–	–	–	–	–	–	–	–	–	–	–	–
Measles	Positive	1	–	126	9	12	–	–	–	–	–	–	–	–	0	–	0	–
	Negative	15	–	278	50	42	–	–	–	–	–	–	–	–	2	–	2	–
	Insufficient sample	–	–	6	–	–	–	–	–	–	–	–	–	–	–	–	–	–
Rubella	Positive	–	–	–	–	13	–	–	–	–	–	–	–	–	–	–	–	–
	Negative	–	–	–	–	27	–	–	–	–	–	–	–	–	–	–	–	–
Dengue	Positive	–	–	0	–	–	154	738	47	1	–	0	0	0	4	–	1	–
	Negative	–	–	2	–	–	309	437	473	12	–	2	2	1	6	–	9	–
	Equivocal	–	–	–	–	–	28	–	32	–	–	–	–	–	–	–	–	–
Chikungunya	Positive	–	–	–	–	–	33	–	217	–	–	–	–	–	–	–	–	–
	Negative	–	–	–	–	–	118	–	372	–	–	–	–	–	–	–	–	–
	Equivocal	–	–	–	–	–	18	–	–	–	–	–	–	–	–	–	–	–
Encephalitis	Positive	–	–	19	–	–	145	2	0	209	0	10	6	1	–	–	2	–
	Negative	–	–	83	–	–	494	119	27	627	161 (81 pooled)	24	28	16	–	–	34	–
	Equivocal	–	–	–	–	–	6	–	–	11	–	–	–	–	–	–	–	–
Hepatitis	Positive	–	–	–	–	–	–	–	–	–	–	–	–	–	229	1	515	19
	Negative	–	–	–	–	–	–	–	–	–	–	–	–	–	444	5	600	21

Dengue and hepatitis-E virus were diagnosed significantly more among males and chikungunya was more in females. Results for the other conditions did not demonstrate any statistically significant difference between the genders. The details are provided in Table 3.

**Table 3.** Difference between the gender distribution of confirmed cases of viral outbreaks between 2010 and 2019 in the state of Odisha

Diagnostic test (IgM)	Result	Gender		*χ*^2^ value	Significance
		Male	Female		
Measles	Positive	70	102	2.64	0.10
	Negative	198	214		
Varicella	Positive	125	140	0.008	0.92
	Negative	78	89		
HSV-1	Positive	6	4	0.55	0.45
	Negative	12	14		
HSV-2	Positive	3	3	0	1
	Negative	15	15		
JE	Positive	106	104	2.55	0.10
	Negative	287	363		
Rubella	Positive	12	13	0.24	0.61
	Negative	27	37		
Dengue	Positive	592	480	14.1	<0.01
	Negative	730	799		
Chikungunya	Positive	98	166	6.43	0.01
	Negative	417	491		
HAV	Positive	124	109	0.72	0.39
	Negative	256	196		
HEV	Positive	307	211	6.5	0.01
	Negative	334	311		

Majority of the cases reported as well as confirmed of measles, viral encephalitis, chickenpox and rubella were among children and the majority of viral hepatitis, dengue and chikungunya cases were among adults. The age distribution of the confirmed cases is given in Table 4.

**Table 4.** Age distribution of suspected and confirmed cases of viral outbreaks between 2010 and 2019 in the state of Odisha

Age group	Clinically suspected cases (Lab confirmed)							Total
	Chickenpox	Chikungunya	Dengue	Measles	Rubella	Viral encephalitis	Viral hepatitis	
Less than 1 year	1 (0)	0 (0)	2 (2)	3 (3)	0 (0)	15 (3)	0 (0)	21
1–5 years	35 (17)	8 (2)	82 (76)	118 (51)	3 (2)	264 (65)	68 (50)	578
6–12 years	169 (107)	35 (13)	208 (165)	227 (90)	20 (18)	310 (107)	235 (190)	1204
13–18 years	60 (44)	27 (7)	199 (107)	55 (16)	10 (3)	56 (12)	130 (87)	537
19–60 years	146 (91)	431 (195)	1210 (678)	66 (24)	27 (2)	269 (60)	741 (418)	2890
Over 60 years	10 (8)	92 (47)	84 (43)	5 (1)	0 (0)	16 (6)	49 (26)	256
Total	421 (267)	593 (264)	1785 (1071)	474 (185)	60 (25)	930 (253)	1223 (771)	5486
% Positive	63.4%	44.5%	60.0%	39.0%	41.6%	27.2%	63.0%	–

**Fig. 3.**
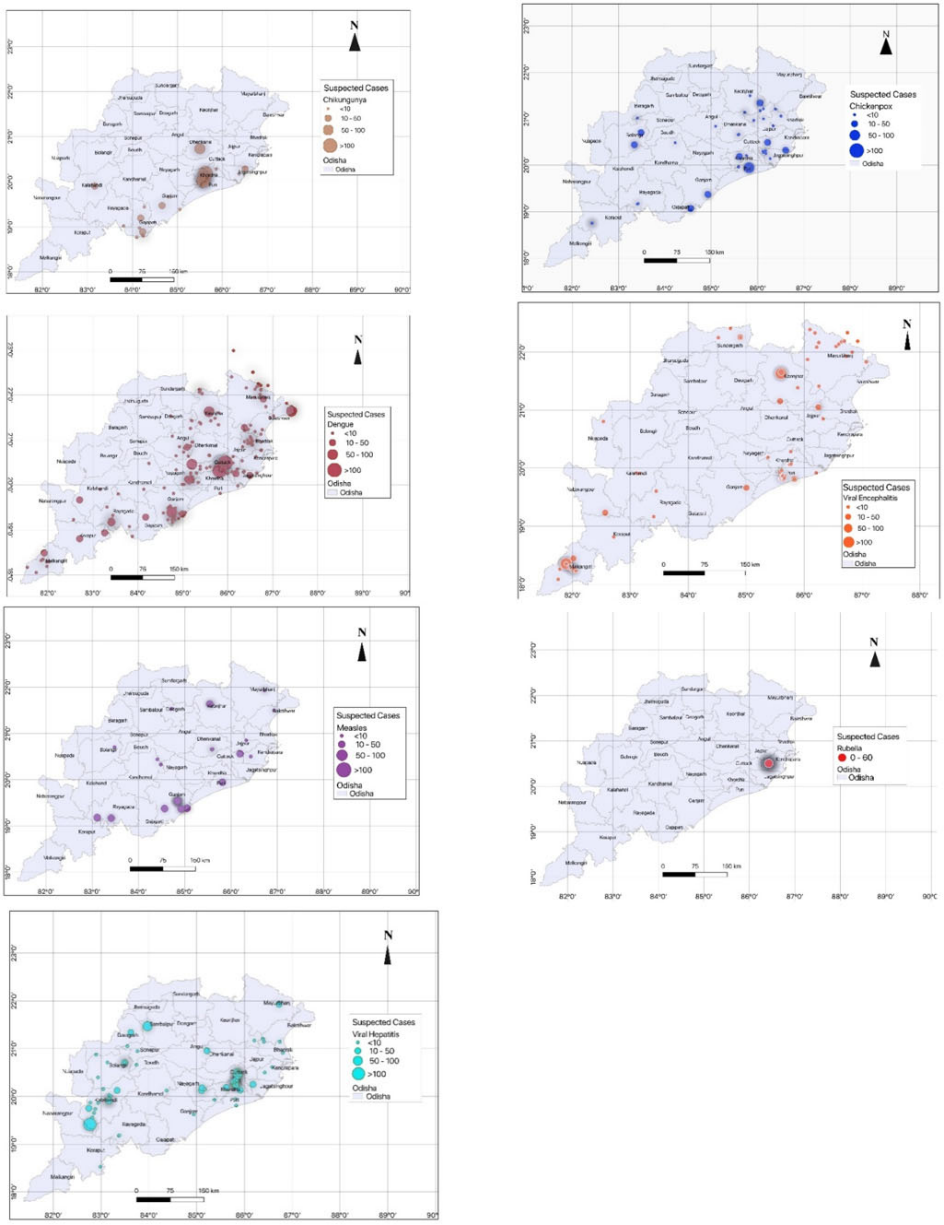
Geographical distribution of the viral outbreaks between 2010 and 2019 in the state of Odisha.

There were regional variations in the types of outbreaks. Most of the outbreaks were limited to the coastal plains of the state. Viral hepatitis outbreaks were distributed in both coastal and western parts of the state. The geographical distribution of each of the outbreaks is shown in Figure 3.

## Context of specific diseases in the region

### Dengue

Dengue is the second most common type of outbreak in Odisha, reported from almost all the districts. Odisha has reported 45 dengue outbreaks; involving 1185 cases and the number of cases has been rising steadily. The peak number of cases and outbreaks were found in the year 2013–14. Dengue outbreaks also show seasonal trends with maximum positive cases reported in the month of July to October. The mean age of the suspected cases for dengue was 30.3 years (95% CI 29.5–31.1). It was also found that males were more commonly affected than females. Dengue was confirmed by testing for ELISA NS1 and IgM antibody. Serotyping was done through RTPCR by amplifying and dengue serotype 2 was the most common serotype found during 2010–2011. Thereafter, all four serotypes 1, 2, 3, 4 were detected during 2012–2017.

### Chikungunya

Most of the chikungunya outbreaks occurred in the year 2006 and since then no chikungunya outbreak has occurred till 2019. The common age group affected is 19–60 years (72.7%) with a female preponderance (62.8% of positive cases).

### Viral hepatitis

Viral hepatitis was found to be the most common type accounting for a total of 55 outbreaks involving 1223 cases in Odisha. These were caused by hepatitis A viruses (HAV) (30.1%) and hepatitis A viruses (HEV) (69.9%). Viral hepatitis most commonly affected the adult age group of 19–60 years with a significant association with gender and a male preponderance [17,18]. The reasons for such male preponderance are not clearly understood.

### Chickenpox

Around 30 chickenpox outbreaks occurred in Odisha involving 421 cases in which the history of varicella zoster virus (VZV) vaccination of the cases was absent or could not be assessed/documented. As the chickenpox vaccination is not covered in the universal immunisation programme, a section of the population remains susceptible to varicella zoster infection, more so during the outbreaks.

### Viral encephalitis

Viral encephalitis can be caused by a number of viruses like herpes simplex virus (HSV), VZV, Epstein–Barr virus, enterovirus, HHV-6 and arboviruses. Most common aetiology for outbreaks of viral acute encephalitis in the state was found to be due to Japanese encephalitis followed by dengue, HSV and measles. Two cases of HEV infection also had encephalitis as a major symptom. There is variation in the causes of the viral encephalitis reported from various studies. Acute encephalopathy syndrome (AES) outbreaks were seen among the tribal population of southern and northern Odisha. A major vaccination drive for Japanese encephalitis virus (JEV) was undertaken by the National Vector Borne Disease Control Program (NVBDCP) in the year 2017 after which the cases of Japanese encephalitis has significantly decreased.

### Measles and rubella

All the samples from an outbreak with a history of fever, macular rash with or without rhinorrhoea and diarrhoea were tested for measles as well as rubella. If found negative they were tested for dengue and scrub typhus as per the algorithm for diagnosing a case of fever with rash. About 23 outbreaks of measles involving 464 cases occurred in the state with only one outbreak of rubella involving around 60 cases from a single district. Majority of measles and rubella cases affected the children under 12 years of age. Measles vaccination drive was systemically implemented throughout India from 2010. The combination vaccination of measles and rubella was introduced in the universal immunisation programme from the year 2015. In the year 2018–2019, about 11 million children were vaccinated with the MR vaccine between the age group of 9 months to 15 years.

## Discussion

The study summarises findings of 191 outbreak investigations spread across a decade in the eastern state of India and was based out on epidemiological investigations and laboratory diagnostics of the VRDL. The study found that the epidemiology of viral outbreaks is variable across regions of the state, with different target populations, seasonal trends and geospatial distributions. Outbreak investigation, as well as management, needs a coordinated network of public health and clinicians for screening of cases, investigation and management. Timely response to disease outbreaks prevents loss of life and wastage of resources.

Viral transmission involves complex systems that include interactions between humans, animals and the environment, and understanding the interactions between human, animal and environmental systems, and the processes within each of the systems, is critical for efficient prevention and minimisation of viral outbreaks.

An outbreak is defined as more cases of a disease than expected in a specific location over a specific period [3]. Worldwide, it is seen that the frequency of reported outbreaks of infectious diseases has increased manifold and the same trend is evident in India and the eastern state of Odisha [5–11].

The state of Odisha is working in close coordination with the outbreak response team of VRDL, unit of ICMR-RMRC, Bhubaneswar, consisting of clinicians, microbiologists, epidemiologists and laboratory technicians and working towards outbreak investigations since 2010 till date. This study focuses on 191 outbreaks caused by different viruses between September 2010 and September 2019. These viral outbreaks have occurred in all 30 districts of Odisha. There remain significant geo-climatic differences between regions of the state affected with outbreaks due to different viral diseases.

A total of 5486 suspected cases were included in these investigations, among them around half were male. The mean age of the study participants was 26.7 years (s.d. = 18.3 years) and majority of the participants were adults between 18 and 60 years of age (2890; 52.7%) followed by children between 5 and 13 years (1204; 21.9%).

Our findings show that the most commonly suspected (and confirmed) aetiologies of the outbreaks were viral hepatitis, followed by dengue. According to IDSP data, in Odisha, a majority of outbreaks during the same period were due to diarrhoeal diseases followed by food poisoning and measles [19]. The current study, while focusing on viral outbreaks alone, captured data from the IDSP reporting structure as well as other sources of information on outbreaks. Hence, it was more comprehensive in efforts to capture suspected outbreaks.

Viral hepatitis was found to be the most common type of outbreak, and HEV and HAV together accounted for all the cases. Majority of the studies in India have shown that outbreaks due to viral hepatitis are caused by HAV (12.6%) and HEV (16.1%) out of the total viral hepatitis cases reported [20]. We found viral hepatitis to be most commonly affecting the adult age group of 19–60 years. However, according to a systematic review of studies from Africa, the most common age group affected was children and adolescents [21]. This review was, however, in an endemic region and used sero-surveillance data. There is evidence of changing epidemiological characteristics of HAV infection with a decrease in incidence and endemicity of infection [22]. There was a male preponderance for hepatitis in our study. Separate studies from Korea have shown both lack of significant association with gender and male preponderance [17,18]. The reason for such male preponderance is not clearly understood.

Dengue was the second most common type of outbreak involving 1185 cases. This is also seen in previous studies and reports which show an alarming increase of dengue-positive cases from the state [23]. Outbreaks of dengue in the region are seen more frequently in the post-monsoon phase [23,24]. The increase in water logging and the increase in vector index post monsoon may be attributed to this increase. The mean age of the suspected cases for dengue was 30.3 years. According to studies in Odisha, the most common age group affected by dengue infection is 11–20 years[24]. Other studies in India have reported that the mean age group for dengue infection is 22 years. In a few of these studies, the mean age group was found to be less than 15 years [25]. There seems to be a difference in the age distribution pattern between routinely reported sero-surveillance and outbreaks. It was also found that males were more commonly affected than females. In a study done in north India, it was seen that more males are dengue-positive than females and the ratio of male:female to dengue infection to be 1.67 [26]. The sex predilection may be due to the type of diurnal activity of the males and the difference in exposure to the adult mosquito population [26].

Chickenpox, also known as varicella zoster infection, is caused by VZV of the herpes group of viruses. The population of India acquires antibodies due to infection in early childhood or by vaccination. Around 30 chickenpox outbreaks occurred in Odisha involving 421 cases were reported. Similar chickenpox outbreaks have been reported in rural belts of north, parts of west and other parts of India [27–29]. The most common age group affected was 6–12 years. Other studies assessing similar outbreaks have reported the most common age group affected by chickenpox to be less than 5 years (10.3%) followed by 6–10 years (12.3%) [30]. Studies from India and south-east Asia have found an increasing trend in sero-prevalence with age, where16% of children aged less than 5 years, 54% of those aged between 5–14 years and 72% of those aged 15–25 years had been infected [31].

Dengue and chikungunya have the same vector and the geo-climatic predispositions for transmission of disease. However, the number of outbreaks of chikungunya (10 outbreaks) that occurred in the state is far less than dengue (45 outbreaks) during 2010–19. Adults between 19 and 60 years were more commonly affected and a female preponderance was found. A similar study on outbreaks of chikungunya in south India showed that the most commonly affected age group was 5–15 years followed by 15–44 years and chikungunya was also found to be positive in more number of females (14.8%) than in males (9.9%) [32]. It was also seen that most of the outbreaks occurred in the year 2006 and since then less number of chikungunya outbreaks have occurred throughout India [33]. The female dominance for chikungunya cannot be explained and more analytical studies may be required to find out the reasons.

The most common aetiology for outbreaks of viral acute encephalitis in the state was found to be due to Japanese encephalitis followed by dengue, HSV and measles. There is variation in the causes of viral encephalitis reported from various studies. Studies of outbreaks in northern India have found that JEV was the cause in less than 10% cases of AES, and the majority cause remained unexplained [34]. Other studies have found that the most common aetiology found in viral encephalitis is HSV infection [35]. Another study from a rural setting in India concluded that the most common aetiology for viral encephalitis was enteroviruses (11.2%) followed by flavivirus (5.2%), VZV (1.9%) and HSV 1 and 2 (0.6%) [36]. Viral encephalitis is predominant in all the age groups from less than 12 years age group as well as of more than 18 years age group. The mean age group affected for viral encephalitis was found to be 40.2 years in a study conducted in central India [36]. AES outbreaks were seen among the tribal population of southern and northern Odisha.

About 23 outbreaks of measles occurred in the state with only one outbreak of rubella. The majority of measles and rubella cases affected children under 12 years of age. In a study from western India, it was seen that among measles outbreaks reported in the state, 72.4% affected were in the age group of 0–12 years [37].

The outbreaks were reported throughout the year, but maximum cases were reported in July followed by September. The increase in case load is due to the burden of dengue cases which are incident during monsoon and post monsoon season. In the initial months of monsoon, the humidity is also high which promotes the breeding of mosquitoes. The increased density of vectors, i.e. *Aedes egypti* and *Aedes albopictus*, during these months in the region may be responsible for more number of cases [38].

Geospatial distribution of the outbreaks was not uniform across the state. While most of the outbreaks were more in coastal plains of the state, which are more densely populated, viral hepatitis outbreaks were distributed in both coastal and western parts of the state. Encephalitis outbreaks, however, were more concentrated in the mountainous tribal regions in the south and the north. Dengue outbreaks were similarly more in the urban areas of the state.

The study relies on the outbreak investigations carried out by the team of VRDL, and the team members involved in these investigations have not been constant due to obvious reasons of attrition of staff, changing responsibilities and long duration of data presented. However, we have tried to mitigate this limitation by regular capacity building of the involved staff and using a standard template for carrying out investigations and reporting the findings.

## Conclusion

To our knowledge, this is the first ever study that attempts to provide a comprehensive picture of the state of viral disease outbreaks in the region by consolidating data from the last decade. Viral outbreaks are reported throughout the year, but the monsoon and post monsoon period witness more frequent and larger outbreaks, largely due to arboviral diseases or chances of food contamination. The study would help the state health department and program implementers in a great way in planning and implementing strategies for effective management and control of these outbreaks. Efforts may be more focused on the densely populated geographical pockets in the coastal region of the state as they report viral outbreaks more frequently. Efforts to improve JEV vaccine coverage and vigilance for AES outbreaks need to be maintained in the tribal-dominated southern and northern districts of the state. Further research needs to be carried out to explore the gender, population subgroups and geographical vulnerability for some of these viral diseases. With an increased incidence of viral zoonoses, there is an impetus to strengthen collaborations between public health, agricultural and environmental departments, which would require One Health approach for effectively to mitigating viral diseases.

## Data Availability

The datasets used and/or analysed during the current study are available on request to the corresponding author.

## Supplementary material

For supplementary material accompanying this paper visit https://doi.org/10.1017/S0950268820001594. click here to view supplementary material
