# Supplementary material for: Epidemiology of viral disease outbreaks in Odisha, India (2010–2019)
Source: Epidemiol Infect. 2020 Jul 16;148:e162. doi: 10.1017/S0950268820001594 (PMC7424601; doi:10.1017/S0950268820001594)
Supplement: Supplementary file 1 [file S0950268820001594sup.zip › S0950268820001594sup001.docx]

**Acute febrile illness** was defined as a patient with fever of 38°C or higher at presentation to ED or history of fever that persisted for 2–7 days with no localizing source.

#### Clinical case definition for measles

#### Any person with fever and maculopapular rash

**WHO definition**

Any person in whom a clinician suspects measles infection, **or**
Any person with fever **and** maculopapular rash (i.e. non-vesicular) **and** cough, coryza (i.e. runny nose) or conjunctivitis (i.e. red eyes)

#### Laboratory criteria for diagnosis

Presence of measles-specific IgM antibodies

#### Case classification

Countries are advised to use the clinical classification scheme until their programmes meet the following two criteria:

- low level of measles incidence;
- access to a proficient measles laboratory

The laboratory classification scheme should be used by countries in the low incidence or elimination phase

#### Clinical classification scheme

**Clinically confirmed:** A case that meets the clinical case definition
**Discarded:** A suspect case that does not meet the clinical case definition

#### Laboratory classification*

**Laboratory classification can also be used for outbreak investigation (see diagram in special aspects section below)*

**Laboratory-confirmed:** A case that meets the clinical case definition and is laboratory-confirmed
**Epidemiologically confirmed:** A case that meets the clinical case definition and is linked to a laboratory-confirmed case
**Clinically confirmed:** A case that meets the clinical case definition and for which no adequate blood specimen was taken
**Discarded:** A suspect case that does not meet the clinical or laboratory definition

# Respiratory WHO surveillance case definitions for ILI and SARI

### ILI case definition

An acute respiratory infection with:

- measured fever of ≥ 38 C°
- and cough;
- with onset within the last 10 days.

### SARI case definition

An acute respiratory infection with:

- history of fever or measured fever of ≥ 38 C°;
- and cough;
- with onset within the last 10 days;
- and requires hospitalization.

Acute respiratory illness (ARI): Sudden onset of respiratory infection symptoms ( cough, sore throat shortness of breath, coryza)

##### **Viral hepatitis WHO case definition**

#### Clinical description

An acute illness typically including acute jaundice, dark urine, anorexia, malaise, extreme fatigue and right upper quadrant tenderness. Biological signs include increased urine urobilonogen and >2.5 times the upper limit of serum alanine aminotransferase

*Note:* Most infections occur during early childhood. A variable proportion of adult infections are asymptomatic

#### Laboratory criteria for diagnosis

**Hepatitis A:** positive for IgM anti-HAV
**Hepatitis B:** positive for IgM anti-HBc or (less desirably) hepatitis B surface antigen (HBsAg)
**Non-A, non-B:** negative for IgM anti-HAV and IgM anti-HBc or (less desirably) HBsAg
*Note:* The anti-HBc IgM test, specific for acute infection, is not available in most countries. HBsAg is often available but is less desirable since it cannot distinguish acute new infections from exacerbation of chronic hepatitis B. Nevertheless, continued HBsAg seropositivity (> six months) is an indicator of chronic infection. For patients with non-A, non-B, the following testing is used for a diagnosis of acute hepatitis C, D or E
**Hepatitis C:** positive for anti-HCV
**Hepatitis D:** positive for IgM anti-HBc or (less desirably) HBsAg plus anti-HDV positive (N.B. only occurs as co-infection or superinfection of hepatitis B)
**Hepatitis E:** positive for IgM anti-HEV

#### Case classification

**Suspected:** A case that is compatible with the clinical description
**Probable:** Not applicable
**Confirmed:** A suspected case that is laboratory-confirmed **or**, for hepatitis A only, a case compatible with the clinical description in a person who has an epidemiological link (i.e. household or sexual contact with an infected person during the 15-50 days before the onset of symptoms) with a laboratory-confirmed case of hepatitis A

**Diarrhoea: WHO definition :** Diarrhoea is the passage of 3 or more loose or liquid stools per day, or more frequently than is normal for the individual. It is usually a symptom of gastrointestinal infection, which can be caused by a variety of bacterial, viral and parasitic organisms. Infection is spread through contaminated food or drinking-water, or from person to person as a result of poor hygiene.

AES **WHO case definition of Acute Encephalitis Syndrome:**

Case Definition of Suspected case:

            -  Acute onset of fever, not more than 5-7 days duration.

            -  Change in mental status with/ without:

- New onset of seizures (excluding febrile seizures)
- (Other early clinical findings – may include irritability, somnolence or abnormal behaviour greater than that seen with usual febrile illness).

**Based on countrywide surveillance data, available in public domain or unpublished data shared by individual researchers, significant Etiological Agents of AES in different parts of India are the following:**

- - *Japanese encephalitis (JEV)*
  - *Orientia tsutsugamushi (scrub typhus)*
  - *Dengue*
  - *Chikungunya*
  - *West Nile*(Reports From Assam & Kerala)
  - *Leptospirosis*(Reports from Assam)
  - *Streptococcus pneumoniae*(~1%);*Neisseria meningitides*(<1%)*; Hemophilus influenzae*(<1%) (Bacterial Meningitis)
  - *Plasmodium falciparum*(Cerebral Malaria)
  - **Herpes simplex virus*
  - **Enteroviruses*(<1%)
  - **Chandipura*(Reports from AP, MH, GJ & BH)
  - **Mycobacterium Tuberculosis*(Tubercular Meningitis)
