# Supplementary material for: Epidemiology of viral disease outbreaks in Odisha, India (2010–2019)
Source: Epidemiol Infect. 2020 Jul 16;148:e162. doi: 10.1017/S0950268820001594 (PMC7424601; doi:10.1017/S0950268820001594)
Supplement: Supplementary file 1 [file S0950268820001594sup.zip › S0950268820001594sup002.docx]

Influx of information and samples to VRDL :

**Influx of samples and information**

District

PHC

RMRC team

Subcentre

Media report/State Health department

Outbreak investigation

(Self collection)

Syndromic diagnostic algorithms:

| Syndrome | Recommended samples | Diseases investigated | Techniques used |
| --- | --- | --- | --- |
| 1. Acute febrile illness with rash | Acute phase serum/plasma | Measles, Rubella, Chikungunya, Dengue, Scrub typhus | IgM ELISA; RT-PCR |
| 1. Acute Respiratory syndrome | Nasopharyngeal swabs, throat swabs, nasal aspirate (with cold chain) | Influenza A & B, Para influenza, Human metapneumo viruses, RSV A & B | PCR, RT PCR, Duplex RT PCR |
| 1. Viral Diarrhea | Stool | Rotavirus, Adenovirus | ELISA, PCR |
| 1. Viral Hepatitis | Serum/ Plasma | HBV, HCV, HAV, HEV | RDK, IgM ELISA, PCR |
| 1. Viral Encephalitis | CSF  Blood/serum,  Plasma | Dengue, JE, Chikungunya, Herpes, Enterovirus, West Nile | IgM-ELISA; DNA PCR; RNA PCR |

Sample analysis: Sample is analysed as per the diagnostic algorithms. These particular algorithms are followed in all the VRDLs of the country.

Suspected AES case

Prompt CSF, Blood /Serum collection.

Prepare 2-3 aliquots

**CSF**

CSF cell count/Biochemistry

JE IgM Assay

Report results to clinicians

**Blood /Serum**

JEV IgM, Scrub Typhus IgM, Dengue NS1/IgM , Chikungunya IgM

Malaria (PS/rDT)

**Serology**

1. West Nile IgM
2. Leptospirosis IgM

**Molecular tests (PCR)**

Herpes virus DNA PCR

Enterovirus RNA PCR

**Bacterial PCR**

*H.infuenzae*

*S.pneumoniae*

*N.meningitidis*

MTB gene expert

Chandipura.

No diagnosis

ELISA positive

**Aliquot 2**

Storage at <(-70֯C)

PCR for:

*Astrovirus,*

*Norovirus &*

*Sapovirus*

**Diarrhoea**

Suspected Viral diarrhoea cases presenting with acute watery diarrhoea with or without vomiting, pain abdomen and signs of dehydration

Report for adenovirus positive

ELISA negative

RNA cDNA synthesis with random hexameric primers

Rotavirus Antigen

(ELISA)

DNA PCR for *adenovirus*

Nucleic acid extraction for non rotavirus

Stool collection

**Aliquot 1**

Prepare 10% stool suspension in PBS (Viral suspension)

Rotavirus positive

Rotavirus negative


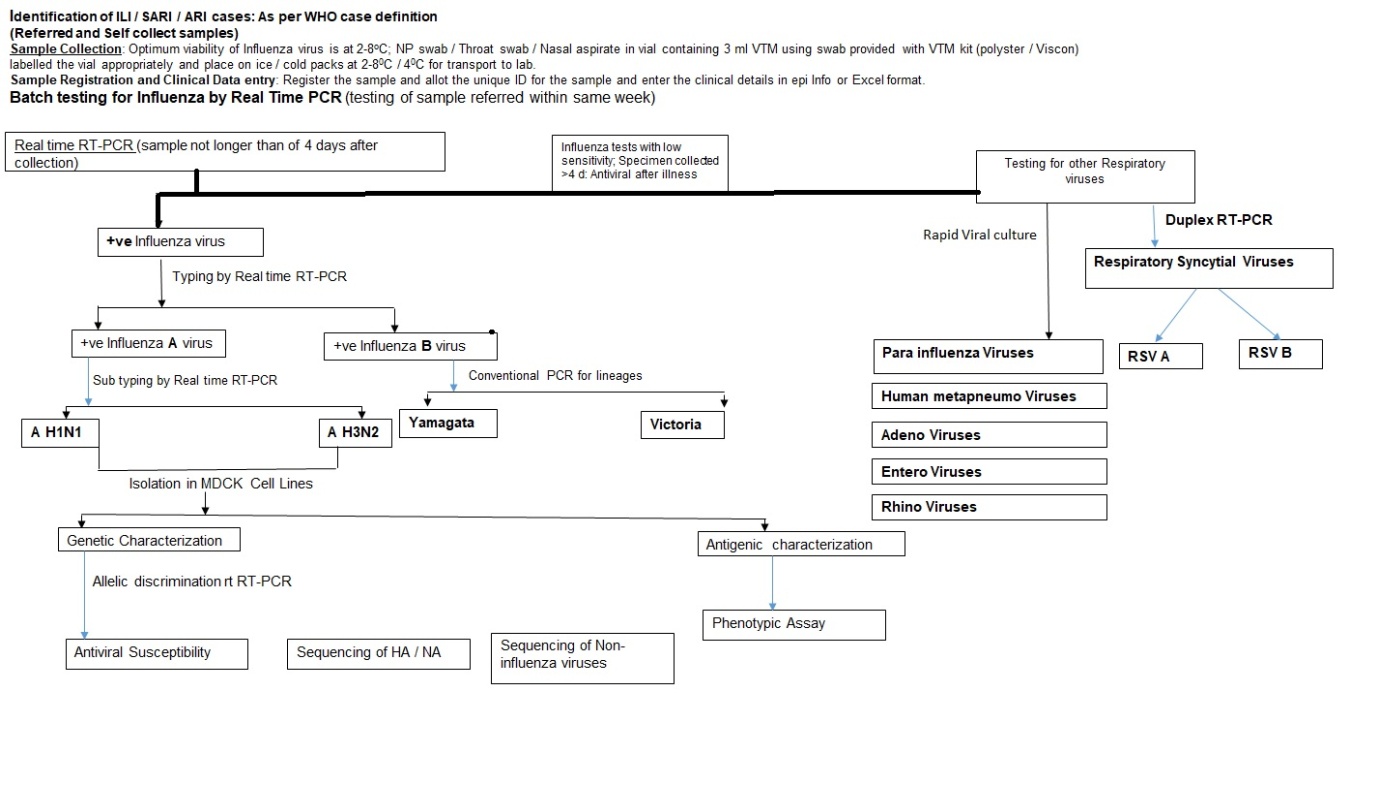


**Diagnosis of Viral Hepatitis in non-jaundice patients**

**Specimen**: Serum/

Plasma

HCV

HBV

Anti HCV

HBsAg

Reactive

Non Reactive

HBV positive

HCV Ab positive

Reactive

Non Reactive

HBV Negative

HCV Ab Negative

**Results for Dengue/Chikungunya Negative**

**EBV IgM**

**Coxsackie IgM**

**HSV I IgM, HSV II IgM**

**Scrub Typhus IgM**

**VZV IgM**

**< 5 Days Of Fever Onset**

**> 5 Days Of Fever Onset**

**Dengue Ns1 Ag**

**Chikungunya PCR**

**Dengue IgM**

**Chikungunya IgM**

**FEVER WITH RASH**

**Measles IgM**

**Rubella IgM**

**Rubella Positive**

**Rubella Negative**

**Measles Positive**

**Measles Negative**

**SAMPLE**
